# Development and validation of a nomogram for predicting early-onset severe intraventricular hemorrhage in extremely preterm infants

**DOI:** 10.3389/fped.2025.1644298

**Published:** 2025-09-01

**Authors:** Yuan Hu, Qin Li, Qin Huang, Ling Yan

**Affiliations:** Department of Pediatrics, The First Affiliated Hospital of Army Medical University, Chongqing, China

**Keywords:** extremely preterm neonates, intraventricular hemorrhage, nomogram, predictive model, clinical utility

## Abstract

**Objective:**

Severe intraventricular hemorrhage (IVH) remains a major complication in extremely preterm infants, with significant clinical implications. We aimed to develop and internally validate a nomogram for forecasting the likelihood of early onset of severe IVH in extremely preterm neonates.

**Methods:**

In this study, a retrospective review of clinical data was conducted among premature infants born before 32 weeks’ gestation who were treated at the pediatric unit of the First Affiliated Hospital of the Army Medical University in Chongqing, China, from January 2017 through December 2023. The group of infants was split randomly into two segments—a training group consisting of 230 individuals and an internal validation group with 98—essentially a 7:3 split. According to the Volpe classification of IVH, the training group was divided into a severe IVH group (Volpe grades III–IV, *n* = 46) and a mild/no IVH group (Volpe grades I–II and no IVH, *n* = 184). Key predictive variables were identified through least absolute shrinkage and selection operator (LASSO) regression. The predictive model's performance was assessed using multiple metrics: receiver operating characteristic (ROC) curve analysis to measure discrimination, calibration plots to evaluate accuracy, and decision curve analysis (DCA) to determine clinical utility.

**Results:**

Six predictors were identified in the training cohort: gestational age, 5-min Apgar score, septic shock, pulmonary hemorrhage, hemoglobin count, and thrombocytes count. The nomogram showed very good performance, yielding an area under the ROC curve (AUC) of 0.877 (95% CI, 0.815–0.939) in the training set and 0.838 (95% CI, 0.712–0.964) in the validation set. Calibration plots showed close agreement with the ideal line, and DCA indicated a substantial net clinical benefit.

**Conclusion:**

This nomogram offers a precise, personalized method for early detection of severe IVH risk in extremely preterm infants, aiding prompt clinical decisions.

## 1 Introduction

Even with significant progress in neonatal intensive care, extremely premature infants (gestational age <32 weeks) still face a high risk of intraventricular hemorrhage (IVH) (1). This enduring challenge is likely associated with increased survival rates, which, while enhancing overall survival, also continue to pose significant risks to neurodevelopment. IVH is the prevalent form of intracranial hemorrhage in premature infants, affecting 25%–40% of very low birth weight (VLBW) neonates (2, 3). The most severe cases (classified as Volpe grades III–IV) (4) make up roughly 10%–15% of these incidents (2, 3). IVH occurrences in VLBW often take place within the first week, peaking during the first three days (5). The primary factors contributing to IVH involve unstable blood flow during the perinatal period, disrupted brain blood flow regulation, and severe swings in oxygen levels (6, 7). Extremely premature infants have underdeveloped blood vessels in the brain, which are delicate and struggle to maintain stable circulation. This makes them highly susceptible to hemorrhage under conditions such as blood pressure instability, hypoxemia, or hypercapnia (8, 9). Additionally, infections and inflammatory conditions—like maternal chorioamnionitis or sepsis in newborns—worsen the risk of IVH by triggering systemic inflammatory responses and compromising the integrity of the blood-brain barrier (10, 11).

One of the most serious outcomes of IVH is damage to white matter, which can lead to both immediate neurological issues and lasting developmental challenges like cerebral palsy and cognitive impairments (1, 12–14). Therefore, it is crucial to develop methods that can directly evaluate the efficacy of interventions. These approaches will establish a clinical basis for effective IVH prevention strategies, such as optimizing hemodynamic management and controlling infection and inflammatory responses, with the overarching aim of reducing IVH incidence and improving long-term effects in extremely preterm newborns.

To address the unmet need for early, actionable risk stratification in extremely preterm infants, this study developed and validated a novel predictive nomogram for severe IVH during the critical first postnatal week. Our approach uniquely combines time sensitive clinical indicators measurable within 48 h after birth, including initial hemoglobin levels and septic shock status, with dynamic physiological parameters and established prenatal/perinatal risk factors into a clinically implementable tool. This comprehensive modeling strategy enables real time risk assessment during the optimal window for early interventions such as hemodynamic optimization and targeted anti-inflammatory therapies. The nomogram provides probabilistic outputs to support clinical decision making, offering guidance for individualized treatment plans, NICU resource allocation, and informed parental counseling, thereby addressing persistent gaps in current neonatal practice.

## 2 Materials and methods

### 2.1 Study design and population

This retrospective cohort study analyzed medical records of all premature newborns born before 32 weeks' gestation who were treated at the pediatric unit of the First Affiliated Hospital of the Army Medical University in Chongqing, China, from January 2017 to December 2023. Inclusion criteria (1): admission to the pediatric unit within 1 h of birth; (2) cranial ultrasound performed within the first 7 days of life. Exclusion criteria: (1) discharge or withdrawal from treatment prior to completion of cranial ultrasound within the first 7 days; (2) prenatal ultrasound evidence of intracranial hemorrhage or ventricular enlargement; (3) presence of severe congenital malformations or hereditary metabolic disorders (e.g., trisomy 21); (4) severe perinatal asphyxia; (5) incomplete clinical data for either the mother or neonate. After applying predefined inclusion/exclusion criteria, 43 cases were excluded, resulting in a final study population of 328 infants. Patients were stratified into two groups based on cranial ultrasound findings: the severe IVH group and the mild/no IVH group.

The study protocol was registered and approved by the Chinese Clinical Trial Registry (NO. ChiCTR2400093198) and was granted approval by the Ethics Committee of the First Affiliated Hospital of Army Medical University (KY2024246), with a waiver of informed consent.

### 2.2 Diagnosis of IVH

The diagnosis and classification of IVH followed the Volpe grading system (4): Grade I (mild), characterized by hemorrhage limited to the subependymal germinal matrix; Grade II (moderate), occured when the hemorrhage spreads into the lateral ventricles but doesn't lead to ventricular enlargement, with less than half of the ventricular area affected; Grade III (severe), marked by hemorrhage that fills over 50% of the lateral ventricles, accompanied by noticeable ventricular expansion; and Grade IV (severe), where hemorrhagic infarction affects the periventricular white matter on the same side as the hemorrhagic lateral ventricle. Bedside cranial ultrasound examinations were performed by sonographers during the first week of life, with subsequent follow-up examinations conducted weekly thereafter. Two NICU doctors independently reviewed all brain ultrasounds (blinded to patient details). If they disagreed, a third senior doctor made the final call. In this study, the children were divided into two groups according to the Volpe classification: the severe IVH group (including only grade III–IV cases) and the mild/no IVH group (including grade I–II cases and no IVH cases).

### 2.3 Data collection

Clinical data for the enrolled infants were extracted from electronic medical records. The selected parameters represent core variables mandatorily documented during NICU admission in our institution. Medical history: gestational age, sex, birth weight, small for gestational age (SGA) status, and *in vitro* fertilization (IVF) status. Prenatal factors: multiple gestation, delivery methods, meconium contamination in the amniotic fluid, premature rupture of membranes, placental disorders (including placental abruption and placenta previa), advanced maternal age pregnancy (maternal age ≥35 years), gestational hypertension and diabetes, intrahepatic cholestasis during pregnancy, antenatal corticosteroid therapy, and chorioamnionitis. Postnatal factors: 1-min and 5-min Apgar scores, presence of grade III–IV neonatal respiratory distress syndrome (NRDS), septic shock, acute respiratory distress syndrome (ARDS), pulmonary hypertension (PPHN), pulmonary hemorrhage, endotracheal intubation in the delivery room, use of invasive mechanical ventilation, and administration of vasoactive drugs within the first 24 h of life. Laboratory data: blood gas analysis within 48 h of birth (pH, PCO₂, PO₂, lactate) and complete blood count within 48 h (white blood cells, hemoglobin, thrombocytes count, neutrophil), along with serum albumin levels. If multiple tests were performed within the first 24 h, the maximum lactate value was recorded, and the minimum values of all other parameters were used. All included cases had complete clinical documentation, as cases with any missing maternal or neonatal records were excluded per our predefined criteria.

### 2.4 Statistical methods

The dataset was divided up into a training set and a validation set in a 7:3 ratio. Variables were compared between the groups. Normally distributed continuous data were expressed as mean ± SD, and non-parametric data as median (P25, P75). Group comparisons were performed with Student's *t* test for normally distributed data or the Mann–Whitney *U* test for non-parametric data. Frequencies and percentages (%) represent categorical variables, and the chi-square or Fisher's exact tests are employed for analyzing group differences. Continuous predictors were standardized to *z*-scores (mean = 0, SD = 1) before analysis to ensure equitable coefficient penalization, while categorical variables retained original binary coding (0/1). For predictive modeling, feature selection was performed using least absolute shrinkage and selection operator (LASSO) regression with L1 regularization. This approach was preferred over stepwise methods due to its superior handling of multicollinearity and overfitting in moderate sized datasets. The optimal penalty parameter (*λ*) was determined through 10-fold cross-validation, effectively shrinking non-informative predictors to zero while preserving clinically relevant variables. Given our events-per-variable (EPV) ratio of 7.7 (46 severe IVH cases/6 predictors), we implemented rigorous safeguards including: (1) bootstrap validation (1,000 iterations) demonstrating minimal optimism (0.02) in performance estimates, and (2) restriction to predictors with established biological plausibility. Variables with non-zero coefficients were subsequently entered into multivariable logistic regression to construct the final nomogram. Model performance was evaluated through: (1) ROC analysis (AUC range: 0.5 = chance to 1.0 = perfect prediction), (2) calibration plots comparing observed vs. predicted probabilities, and (3) decision curve analysis for clinical threshold optimization. All analyses used R v4.2.2 with statistical significance defined as *p* < 0.05. Recent methodological studies support that such regularized models maintain reliability at EPV ≥5 when combined with robust validation (15).

## 3 Results

### 3.1 The characteristics of severe IVH in extremely preterm infants

**Figure 1:**
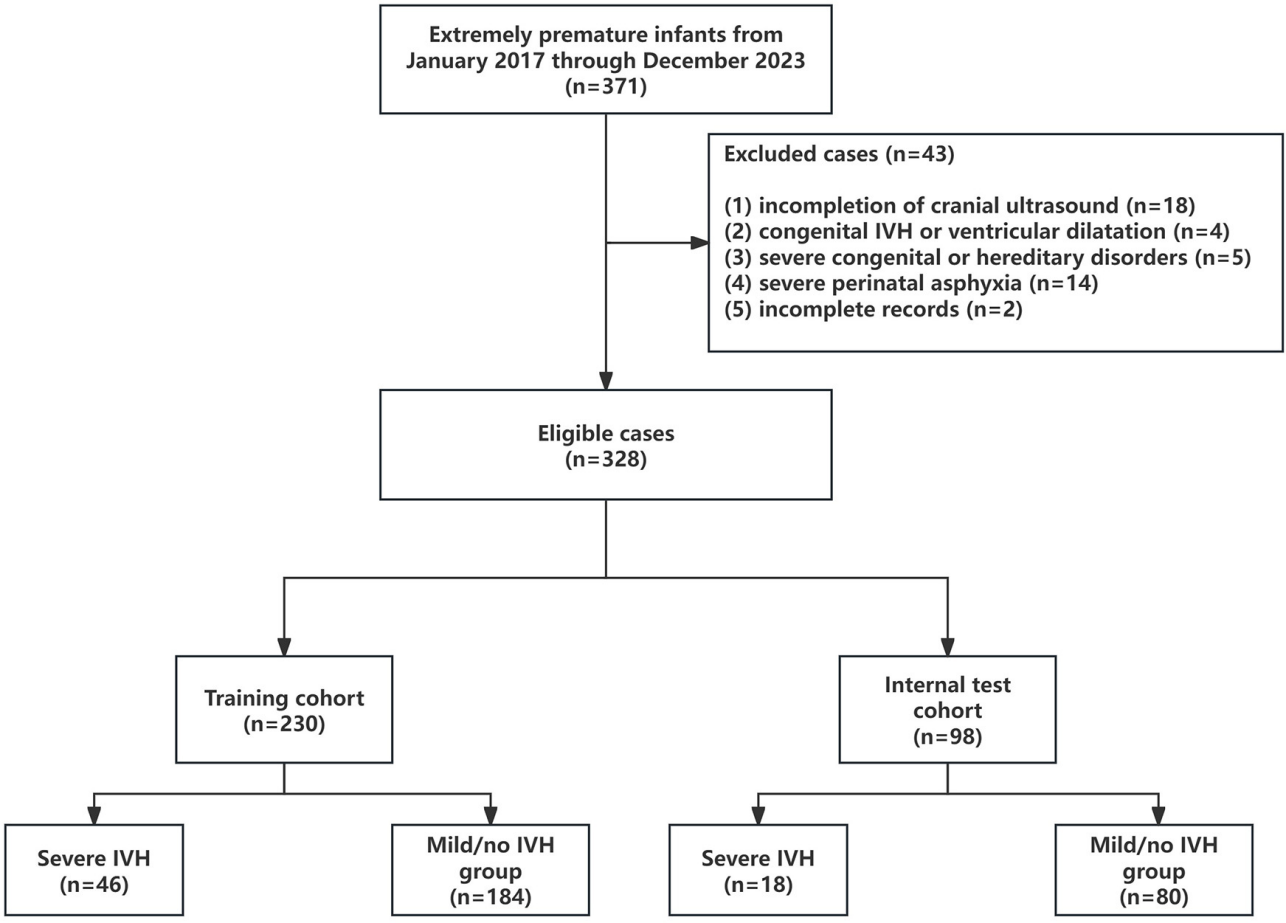
Flow chart for patient selection.

This study involved 328 infants, randomly split into a training cohort (*n* = 230) and an internal test cohort (*n* = 98) at a 7:3 ratio. This ratio was chosen to retain adequate severe IVH cases in both cohorts and balance model complexity (development needs) against performance evaluation (validation needs).The training cohort comprised 46 severe IVH cases and 184 control cases (Figure 1). Table 1 outlines the baseline demographic and clinical characteristics of both cohorts. We acknowledge that the multiple univariate comparisons may increase the risk of Type I errors, though these were exploratory analyses to characterize cohort differences. The baseline features of the training and internal validation groups were generally comparable (*p* > 0.05), with the exception of the proportion of IVF and the prevalence of sepsis shock, indicating that the groups are sufficiently similar for model development and validation. In the training set, 46 of 230 neonates (20%) developed severe IVH. A comparison of clinical characteristics between the severe and mild/no IVH groups (Table 1) showed that neonates with severe IVH had significantly lower gestational ages (203 vs. 215 days, *p* < 0.001) and birth weights (1,144 ± 318 g vs. 1,387 ± 310 g, *p* < 0.001). Male, IVF, and multiple gestations were more common in the severe IVH group, which also exhibited lower 1-min and 5-min Apgar scores. No statistically significant differences were observed between the groups in terms of SGA, cesarean section, meconium-stained amniotic fluid, advanced maternal age pregnancy, gestational hypertension, gestational diabetes mellitus, intrahepatic cholestasis of pregnancy, placental disorders, premature rupture of membranes, chorioamnionitis, or antenatal corticosteroid administration. Regarding complications, the severe IVH group had higher rates of septic shock, ARDS, PPHN, and pulmonary hemorrhage. In terms of treatment, the group with severe IVH exhibited notably higher instances of intubation right after birth, early initiation of invasive mechanical ventilation, and reliance on vasoactive medications within the first 24 h of life. Additionally, comparisons of laboratory parameters revealed that the severe IVH group had higher lactate levels and lower hemoglobin, thrombocytes, and albumin levels. No significant differences were found in pH, PCO2, PO2, white blood cell count, or neutrophil count (*p* > 0.05).

**Table 1:** Patient demographics and baseline characteristics.

Characteristic	Training cohort (*n* = 230)	Internal test cohort (*n* = 98)	*p1*	Mild/no IVH group, (*n* = 184)	Severe IVH, (*n* = 46)	*p2*
Gestational age (days)	213 (203, 219)	212 (205, 219)	0.866	215 (208, 219)	203 (192, 211)	<0.001
Birth weight (g)	1,338 ± 326	1,338 ± 306	0.995	1,387 ± 310	1,144 ± 318	<0.001
Male, (yes, %)	131 (57.0%)	54 (55.1%)	0.757	98 (53%)	33 (72%)	0.024
IVF, (yes, %)	29 (12.6%)	28 (28.6%)	<0.001	19 (10%)	10 (22%)	0.037
Multiple gestations, (yes, %)	61 (26.5%)	31 (31.6%)	0.346	42 (23%)	19 (41%)	0.011
SGA, (yes, %)	15 (6.5%)	6 (6.1%)	0.892	12 (7%)	3 (7%)	>0.999
Cesarean section, (yes, %)	145 (63.0%)	63 (64.3%)	0.831	116 (63%)	29 (63%)	>0.999
MSAF, (yes, %)	4 (1.7%)	3 (3.1%)	0.431	3 (2%)	1 (2%)	>0.999
1-min Apgar score	9.00 (7.00, 10.00)	9.00 (7.00, 10.00)	0.419	9.00 (8.00, 10.00)	8.00 (5.00, 9.00)	<0.001
5-min Apgar score	10.00 (9.00, 10.00)	10.00 (9.00, 10.00)	0.725	10.00 (9.00, 10.00)	9.00 (7.00, 10.00)	<0.001
Advanced maternal age pregnancy, (yes, %)	46 (20.0%)	23 (23.5%)	0.480	36 (20%)	10 (22%)	0.742
Gestational hypertension, (yes, %)	61 (26.5%)	23 (23.5%)	0.562	47 (26%)	14 (30%)	0.501
Gestational diabetes mellitus, (yes, %)	52 (22.6%)	21 (21.4%)	0.814	46 (25%)	6 (13%)	0.083
Intrahepatic cholestasis of pregnancy, (yes, %)	9 (3.9%)	8 (8.2%)	0.112	8 (4%)	1 (2%)	0.692
Placental disorders, (yes, %)	22 (9.6%)	13 (13.3%)	0.320	18 (10%)	4 (9%)	>0.999
Premature rupture of membranes, (yes, %)	90 (39.1%)	35 (35.7%)	0.560	71 (39%)	19 (41%)	0.736
Chorioamnionitis, (yes, %)	23 (10.0%)	10 (10.2%)	0.955	15 (8%)	8 (17%)	0.094
Antenatal corticosteroid therapy, (yes, %)	173 (75.2%)	75 (76.5%)	0.800	138 (75%)	35 (76%)	0.879
Grade III-IV NRDS, (yes, %)	45 (19.6%)	21 (21.4%)	0.700	32 (17%)	13 (28%)	0.096
Septic shock, (yes, %)	15 (6.5%)	13 (13.3%)	0.045	5 (3%)	10 (22%)	<0.001
ARDS, (yes, %)	42 (18.3%)	22 (22.4%)	0.381	28 (15%)	14 (30%)	0.017
PPHN, (yes, %)	18 (7.8%)	10 (10.2%)	0.481	10 (5%)	8 (17%)	0.013
Pulmonary hemorrhage, (yes, %)	27 (11.7%)	11 (11.2%)	0.894	12 (7%)	15 (33%)	<0.001
Neonatal intubation in the delivery room, (yes, %)	75 (32.6%)	32 (32.7%)	0.994	47 (26%)	28 (61%)	<0.001
Initial invasive ventilation, (yes, %)	101 (43.9%)	50 (51.0%)	0.237	65 (35%)	36 (78%)	<0.001
Use of vasoactive medications within the first day of life, (yes, %)	56 (24.3%)	23 (23.5%)	0.865	37 (20%)	19 (41%)	0.003
Ph	7.31 (7.24, 7.37)	7.32 (7.24, 7.37)	0.515	7.31 (7.25, 7.37)	7.31 (7.20, 7.38)	0.653
PCO2 (mmHg)	42 (33, 49)	40 (30, 48)	0.300	40 ± 15	42 ± 12	0.221
PO2 (mmHg)	70 (70, 73)	70 (69, 70)	0.542	70 (70, 73)	70 (70, 70)	0.977
Lactate (mmol/L)	1.60 (1.10, 2.78)	1.85 (1.40, 3.28)	0.068	1.50 (1.00, 2.40)	2.45 (1.45, 4.40)	<0.001
White blood cells (×10^9^/L)	8 (6, 12)	7 (6, 11)	0.567	8 (6, 12)	7 (5, 10)	0.282
Hemoglobin (g/L)	158 (139, 177)	159 (126, 179)	0.596	163 ± 29	129 ± 31	<0.001
Thrombocytes (×10^9^/L)	208 (162, 258)	204 (151, 259)	0.592	223 ± 75	170 ± 82	<0.001
Neutrophil (×10^9^/L)	3.7 (2.3, 6.7)	3.7 (2.5, 5.3)	0.879	3.7 (2.3, 6.9)	3.6 (2.0, 6.2)	0.628
Albumin (g/L)	27.9 ± 4.3	27.0 ± 4.9	0.128	28.4 ± 4.0	25.8 ± 4.8	0.002

Exploratory *p* values, interpret with caution due to multiple testing, *p1* for comparison between training cohort and internal test cohort, *p2* for comparison between severe IVH group and mild/no IVH group in training cohort, placental diseases including placental abruption and placenta previa. IVH, intraventricular hemorrhage; IVF, *in vitro* fertilization; SGA, small for gestational age; MASF, meconium-stained amniotic fluid; NRDS, neonatal respiratory distress syndrome; ARDS, neonatal acute respiratory distress syndrome; PPHN, pulmonary hypertension.

### 3.2 Screening of predictive factors using LASSO regression

**Figure 2:**
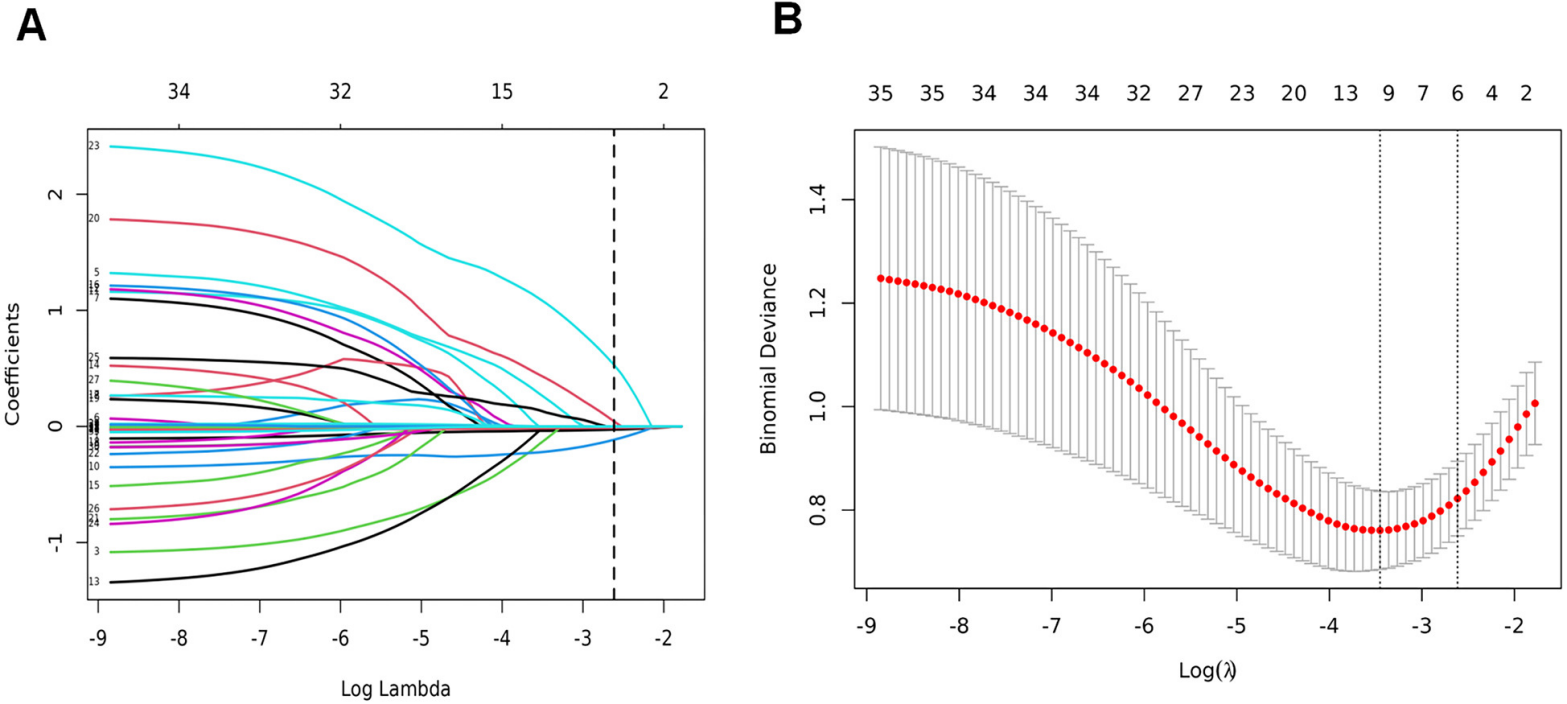
LASSO binary logistic regression model for clinical indicator selection. **(A)** The LASSO model underwent five-fold cross-validation using minimal criteria to identify the optimal parameter (lambda); **(B)** coefficient trajectories for all seven features were visualized across a range of log(lambda) values.

**Figure 3:**
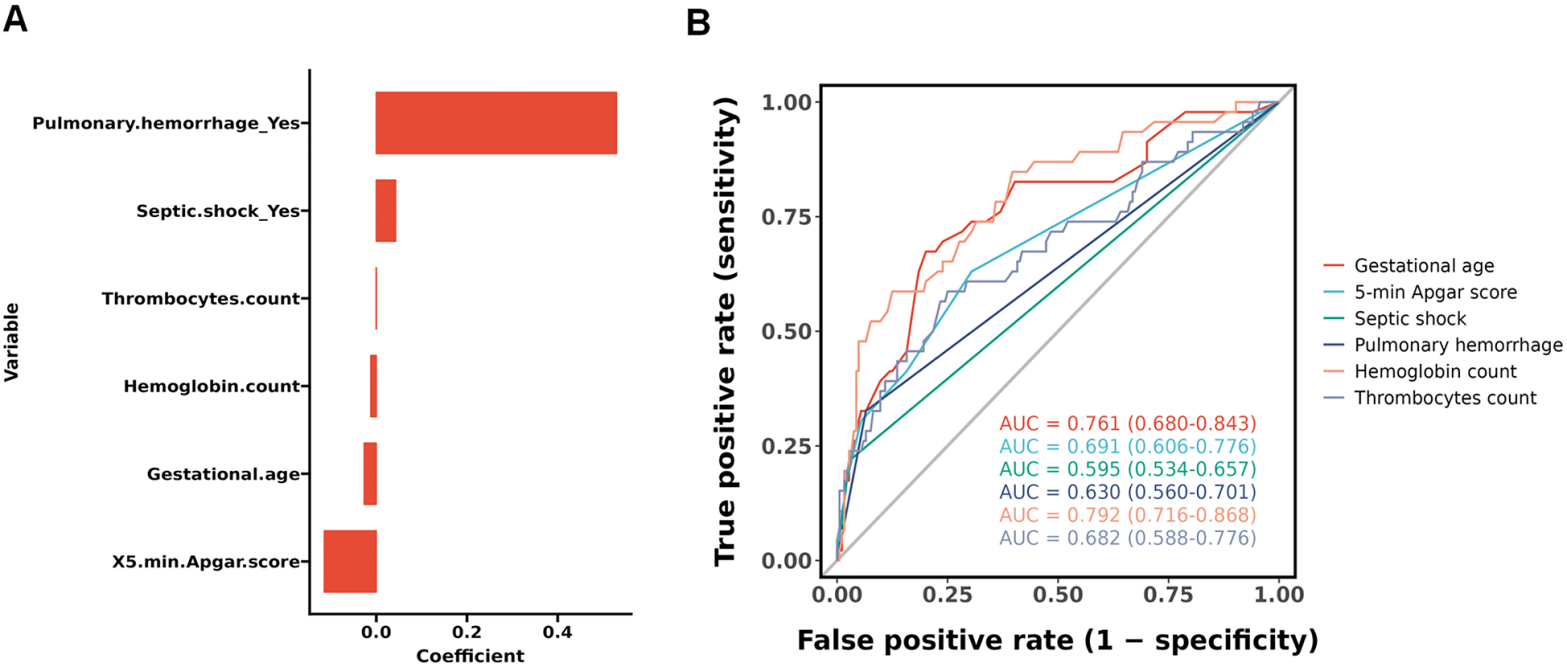
Screening of predictive factors using LASSO regression. **(A)** Histogram of the coefficients of the selected features; **(B)** a single independent variable was used to make the ROC curve for prediction.

All baseline variables presented in Table 1 (including demographic, perinatal, and early postnatal parameters) were initially included as candidate predictors in the LASSO regression analysis without prior selection. This comprehensive approach ensured no potentially relevant variables were excluded based on clinical assumptions or statistical thresholds. The LASSO algorithm (Figure 2) subsequently identified six predictors with non-zero coefficients: gestational age, 5-min Apgar score, septic shock, pulmonary hemorrhage, hemoglobin level, and platelet count (Figure 3A). Figure 3B shows the ROC curves and AUC scores (0.761, 0.691, 0.595, 0.630, 0.792, 0.682) for the six predictive models.

### 3.3 Construction of the nomogram model

**Figure 4:**
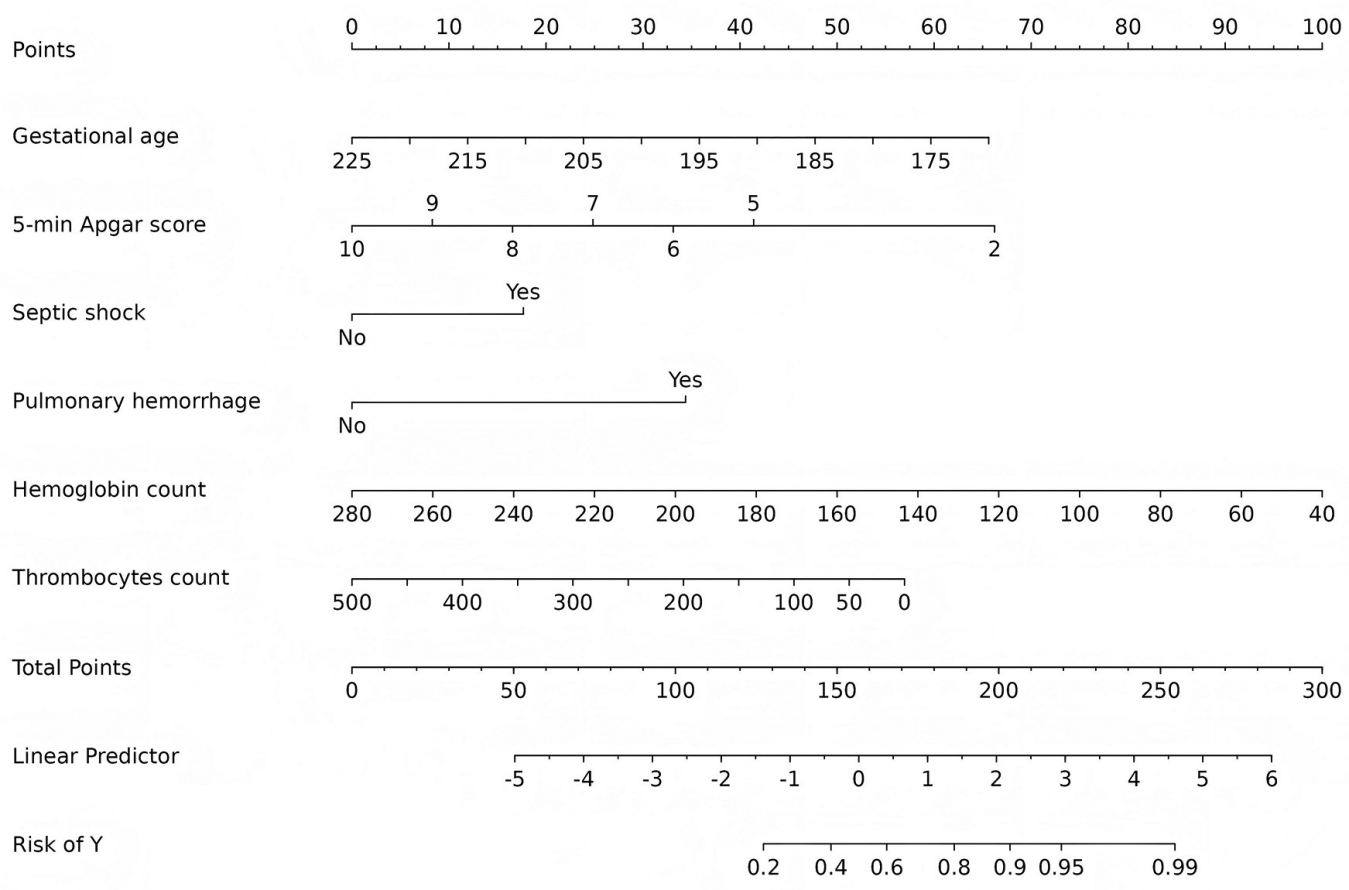
Nomogram models estimating the probability of IVH in extremely preterm infants.

A nomogram was developed using six predictive factors identified through multivariable logistic regression, as shown in Figure 4. The final regression model can be represented by the formula: $\begin{array}{rl} & \ln\;(P/1-P)\;=\;17.309-0.056\;\times (\mathrm{gestational}\;\mathrm{age})-0.389\;\times \\ & \;(5\text{-}\mathrm{minute}\;\mathrm{Apgar}\;\mathrm{score})\;+\;0.831\;\times \;(\mathrm{septic}\;\mathrm{shock})\;+\\ & \;1.617\;\times \;(\mathrm{pulmonary}\;\mathrm{hemorrhage})-0.02\;\times \\ & \left(\mathrm{hemoglobin}\;\mathrm{count})-0.005\;\times \;(\mathrm{thrombocytes}\;\mathrm{count}\right) \end{array}$. In this model, a higher total score reflects an increased risk of developing severe IVH. Consider, for instance, an infant with a gestational age of 205 days and a 5-min Apgar score of 9. This infant experienced septic shock and pulmonary hemorrhage shortly after birth, with a hemoglobin level of 120 g/L and a thrombocyte count of 150 × 10^9^/L, as indicated by routine blood tests. The corresponding scores for each factor are as follows: 24 points for gestational age, 9 points for the Apgar score, 18 points for septic shock, 35 points for pulmonary hemorrhage, 68 points for hemoglobin, and 35 points for thrombocyte, resulting in a total score of approximately 189 points. This score is associated with an estimated probability of 0.85 for the development of severe IVH.

### 3.4 Validation of the nomogram model

**Figure 5:**
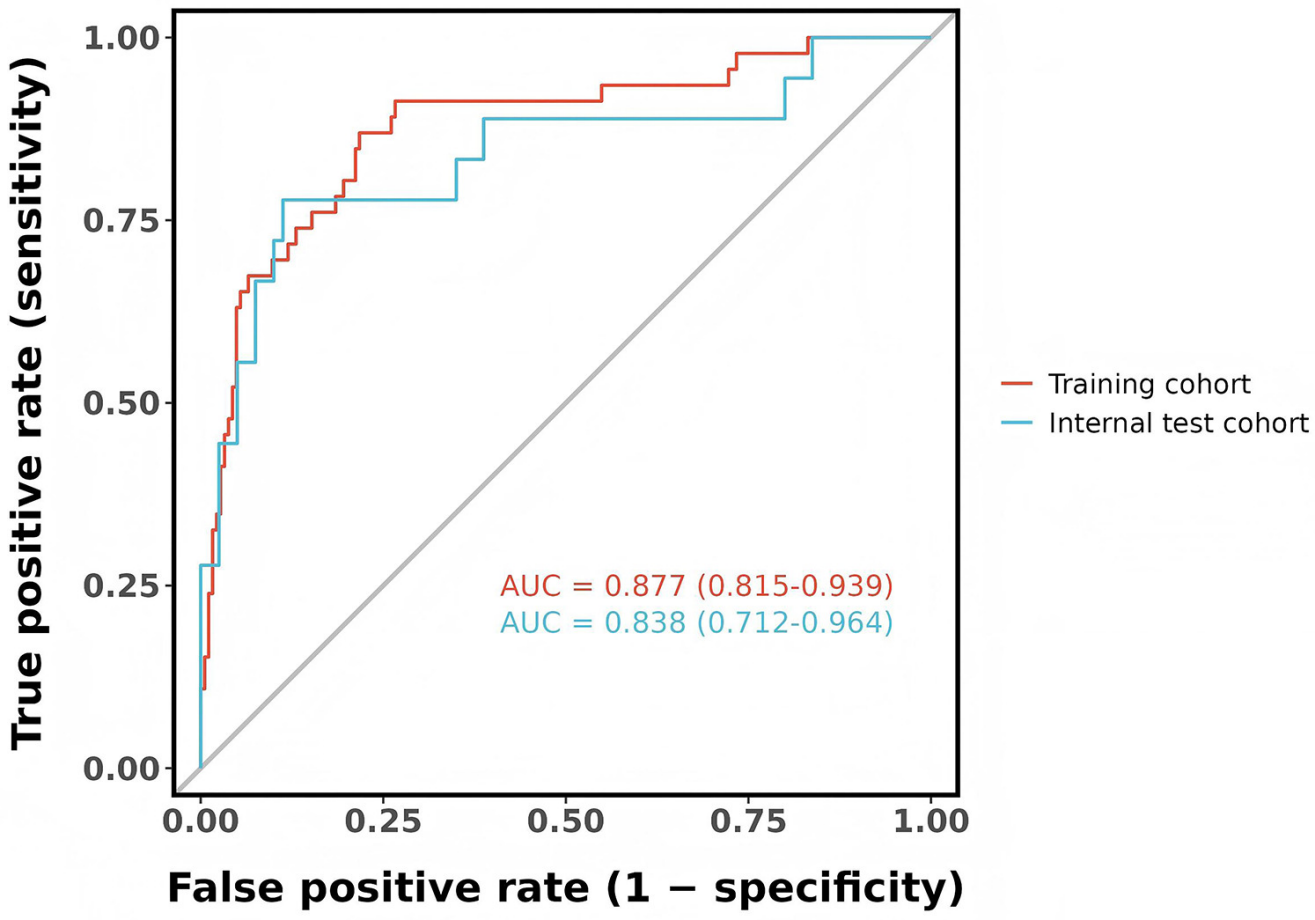
The ROC curves of the nomogram model in training and internal test cohort.

**Figure 6:**
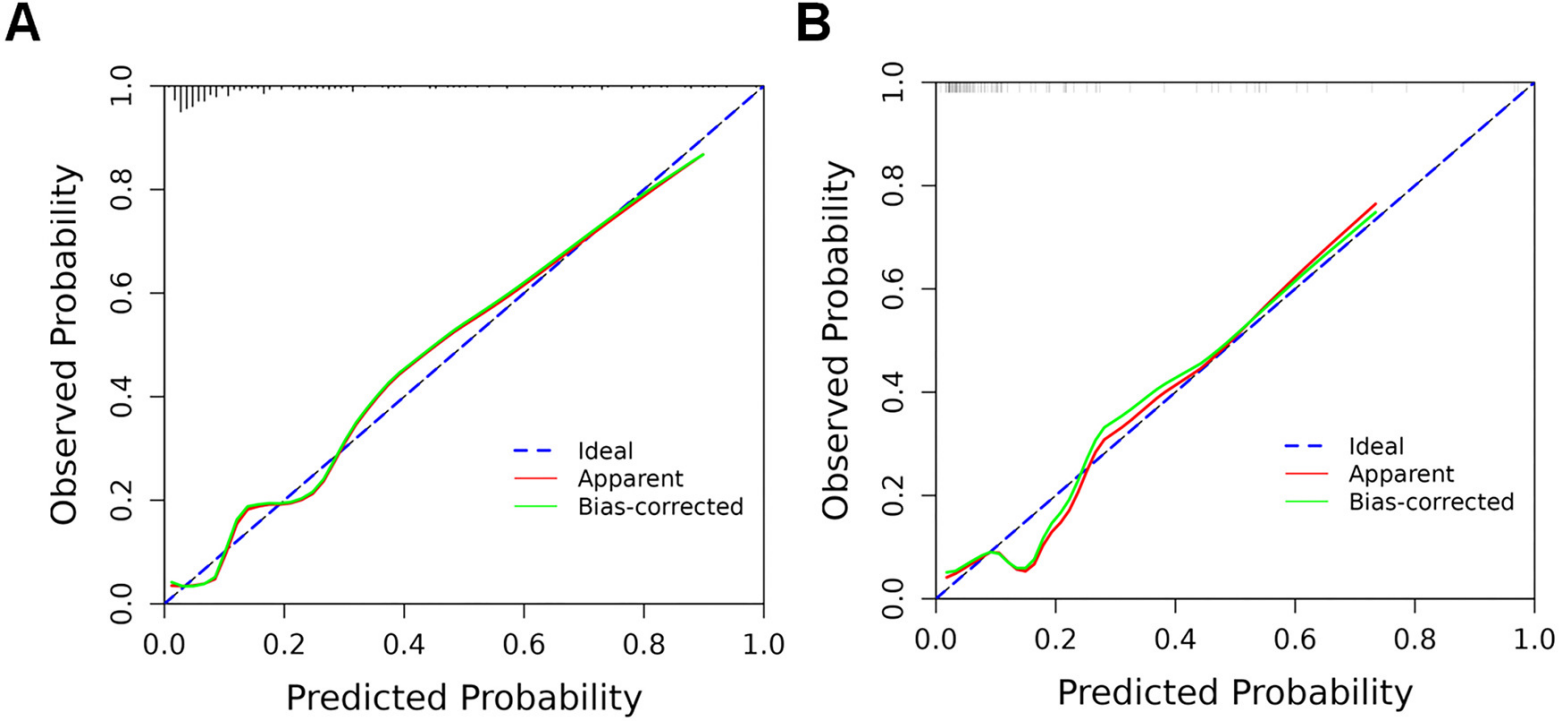
Calibration curves of the nomogram model in training and validation cohorts. **(A)** Training cohort: The calibration curve illustrates the predictive accuracy of the nomogram model; **(B)** validation cohort: The model's performance is similarly depicted in the independent validation set. In both plots, the dashed line indicates the model's actual performance, while the solid diagonal line represents the ideal scenario where predictions perfectly match outcomes.

**Figure 7:**
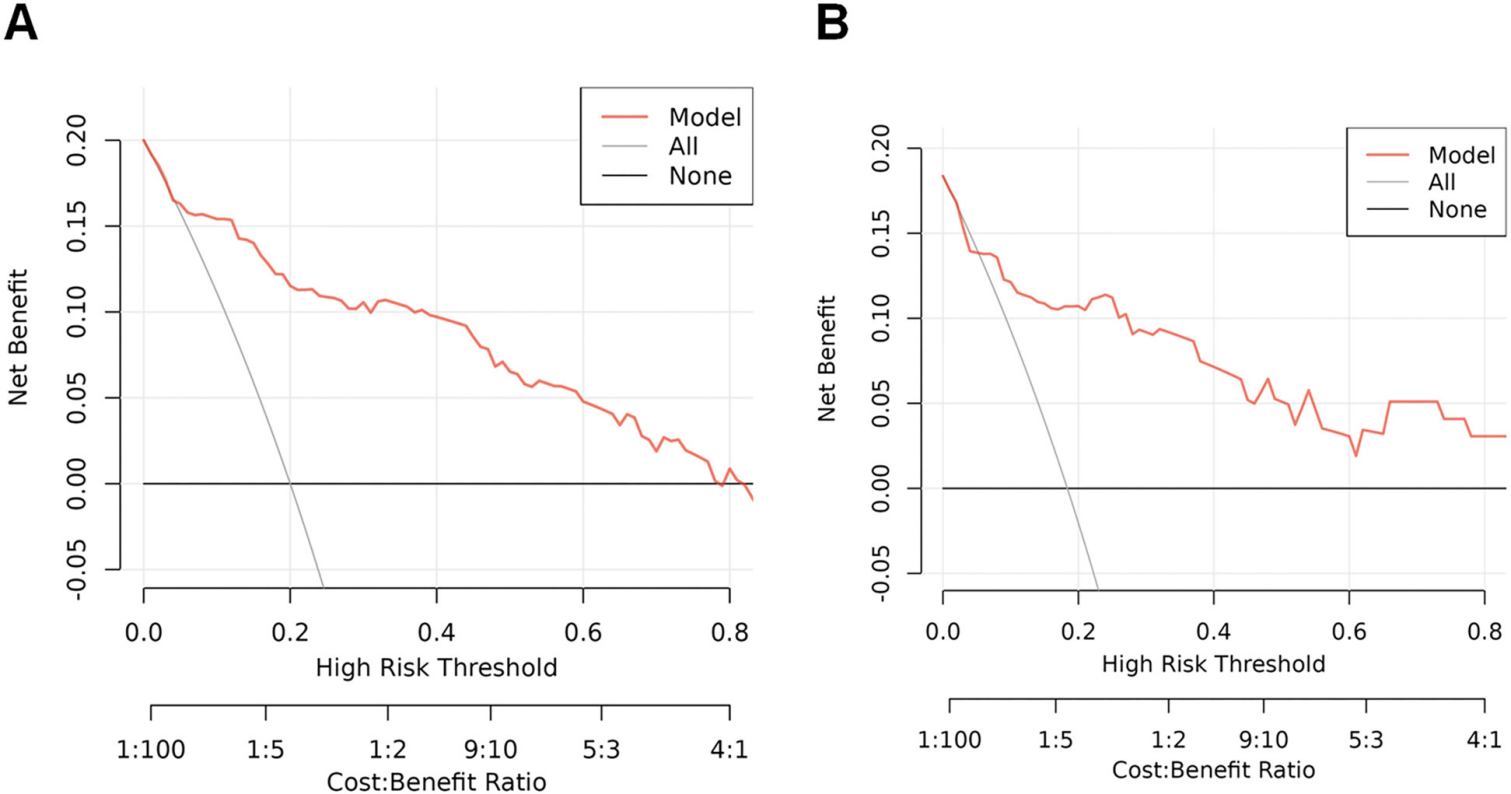
DCA plots and clinical impact curves for the nomogram model in both the training and internal validation cohorts. **(A)** DCA results for the training cohort, illustrating the model's net benefit across various risk thresholds; **(B)** DCA for the internal test cohort, evaluating clinical utility under different decision scenarios. The *y*-axis represents the standardized net benefit, while the *x*-axis indicates the range of risk thresholds and cost-benefit ratios. These curves demonstrate the model's practical value in guiding clinical decision making.

The predictive model's classification accuracy was assessed through ROC analysis. Results showed robust performance, with an AUC of 0.877 (95% CI: 0.815–0.939) for the training dataset and 0.838 (95% CI: 0.712–0.964) for the internal validation set (Figure 5). A bootstrap analysis involving 1,000 replicates revealed excellent calibration in the training group, as evidenced by the close alignment between the calibration curve and the ideal reference line (Figure 6A). This indicates the model's predictions for severe intraventricular hemorrhage probabilities closely match actual outcomes. Figure 7A displays the DCA plot for the training cohort, with the *x*-axis indicating the probability threshold for severe IVH and the *y*-axis showing net benefit. The analysis demonstrated meaningful clinical utility, as the nomogram model provided a strong net benefit across a wide probability range (0.05–0.8). The internal validation cohort's calibration curve closely followed the ideal reference line (Figure 6B), underscoring the model's excellent predictive accuracy. Additionally, the validation cohort's DCA results (Figure 7B) further validated the model's practical value, showing consistent clinical advantages across relevant probability thresholds.

## 4 Discussion

In this study, we constructed a nomogram for forecasting the likelihood of critical IVH in exceptionally premature newborns within their initial life week. Using LASSO and logistic regression, we identified six key variables: gestational age, 5-min Apgar score, septic shock, pulmonary hemorrhage, hemoglobin level, and thrombocyte count. Based on these factors, we constructed a model that effectively assesses the risk of severe IVH, demonstrating robust accuracy and strong discriminatory power. Our findings not only corroborate previously reported associations between perinatal risk factors and IVH, but also underscore the synergistic role of hematological parameters and clinical complications in the pathophysiology of IVH.

A substantial body of evidence establishes a strong association between lower gestational age and an increased risk of severe IVH (16–18). In premature infants, IVH primarily occurs due to underdeveloped blood vessels in the brain. The blood-brain barrier, which relies on a complex network of endothelial cells, tight junctions, basement membrane structures, and astrocyte projections, matures progressively throughout gestation—making its stability directly tied to the infant's stage of fetal development. In preterm birth, insufficient glial fibrillary acidic protein around germinal matrix blood vessels weakens vascular integrity. Additionally, decreased fibronectin levels and reduced collagen content result in a poorly organized basement membrane structure. Downregulation of tight junction proteins (e.g., claudin-5) and aquaporin-4 further exacerbates blood-brain barrier's permeability. These structural and molecular deficits lead to increased vascular fragility, impaired barrier function, and an elevated risk of blood extravasation and hemorrhage (17). Moreover, the underdeveloped smooth muscle layer in cerebral vessels of preterm infants weakens the vasoconstrictive response mediated by α1-adrenergic receptors, impairing the capacity to buffer sudden increases in blood pressure. As a result, cerebral vessels are unable to autoregulate blood flow during hypertensive episodes, transmitting excessive perfusion pressure directly to the fragile capillary network, thereby increasing the risk of vessel rupture. Conversely, hypotension may trigger cerebral ischemia (19, 20). Collectively, these findings suggest that gestational age is not only a biological marker of IVH risk but also a critical determinant for guiding individualized monitoring strategies, such as targeted blood pressure management and restrictive ventilation approaches.

Early postnatal clinical factors are key determinants of IVH in extremely preterm infants. The Apgar score is a commonly employed method for evaluating a newborn's health immediately after delivery. Lower Apgar scores, particularly at 5 min, is closely linked to a higher likelihood of IVH (21–23). This association likely stems from oxygen deprivation related to birth or severe respiratory and circulatory instability, which can trigger oxidative damage and weaken the blood-brain barrier by harming vascular endothelial cells. Additionally, such complications may disrupt the brain's ability to regulate blood flow, increasing the chances of hemorrhage in the germinal matrix (21). These insights highlight the importance of effective resuscitation techniques and close postnatal observation to minimize IVH risk in newborns.

Septic shock and pulmonary bleeding represent intricate medical scenarios that can synergistically contribute to IVH via multiple biological pathways. Research by Luca and colleagues demonstrated a clear link between sepsis—whether confirmed by culture or clinically suspected—and IVH in premature newborns. Advanced statistical modeling revealed sepsis as a standalone predictor of IVH (OR 4.7, 95% CI 1.7–13.1), with the danger becoming particularly pronounced when vasopressor therapy became necessary for circulatory collapse (24). The systemic inflammation characteristic of septic shock compromises the brain's protective barrier, enabling toxic elements to penetrate neural tissue. Simultaneously, the lack of proper control over blood flow to the brain in premature babies makes the white matter particularly susceptible to harm, which can result in issues like cystic periventricular leukomalacia or widespread, non-cystic white matter damage (25, 26). Furthermore, interventions required for sepsis management—such as mechanical ventilation or catecholamine administration—can induce fluctuations in both intrathoracic and intracranial pressures, destabilizing cerebral perfusion (22). Systemic inflammatory response syndrome triggered by sepsis ramps up the production of inflammatory signaling molecules like IL-6 and TNF-α, while simultaneously kicking the coagulation system into overdrive. This cascade of events throws off the body's careful equilibrium between clot formation and breakdown, frequently leading to a dangerous depletion of platelets and the development of disseminated intravascular coagulation—a double whammy that severely compromises the blood's ability to clot properly (27). Pulmonary hemorrhage, frequently accompanied by hypoxemia and hemodynamic instability, indirectly exacerbates fluctuations in cerebral perfusion pressure and increases vascular shear stress, further compromising vascular integrity. These pathological processes are consistent with the mechanisms proposed in neonatal sepsis associated brain injury research.

Our study found that decreased hemoglobin and thrombocyte counts are associated with IVH, emphasizing the pivotal roles of anemia and coagulopathy in its pathogenesis. Christine et al. identified low initial hematocrit as a significant factor linked to IVH, incorporating it as an independent risk variable in their multivariable model (27). Similarly, Siddappa et al. reported that infants with severe IVH exhibited prolonged prothrombin time and international normalized ratio, along with lower thrombocyte counts (28). Furthermore, a predictive model developed by Weinstein et al. demonstrated that higher hematocrit and thrombocyte levels were significantly associated with a reduced incidence of moderate to severe IVH (2). These findings are consistent with previous studies and suggest that maintaining hematologic parameters within normal or elevated ranges may provide a protective effect, while lower levels are linked to an increased risk of IVH.

Given the serious implications of IVH, especially in its more severe stages, creating reliable methods to predict which newborns are at highest risk is crucial for clinical practice. Kumar and colleagues introduced a simple scoring system that factors in gestational age, birth weight, Apgar score at 1 min, place of birth (inborn vs. outborn), and sex—yielding an AUC of 0.77 (29). Meanwhile, Weinstein's team developed a more refined model that includes gestational age, 5-min Apgar score, hemoglobin levels, and platelet count, achieving a stronger predictive accuracy with an AUC of 0.826 (2). Kim et al. employed machine learning algorithms (e.g., XGBoost) and demonstrated that early postnatal data could enhance predictive accuracy (30). However, existing models vary in terms of variable selection, prediction windows, and interpretability. This study employed a comprehensive LASSO regression approach that incorporated all candidate variables for initial screening. This comprehensive strategy capitalizes on LASSO's inherent advantage in handling high dimensional data by automatically shrinking coefficients of irrelevant variables to zero, thereby minimizing subjective selection bias. The selected predictors were subsequently validated through multivariable logistic regression to ensure robustness. The resulting nomogram for predicting early-onset severe IVH in extremely preterm infants demonstrates both strong predictive accuracy and clinical utility. Our predictive model combines six routinely available clinical variables—gestational age, 5-min Apgar score, septic shock, pulmonary hemorrhage, hemoglobin level, and platelet count—to assess severe IVH risk during the critical postnatal period. The model showed significant clinical utility (DCA net benefit range: 0.05–0.8), supporting its use for risk stratification in NICUs. Clinically, this tool enables: (1) intensified neuromonitoring (including serial cranial ultrasounds) for infants with >30% predicted risk, and (2) personalized interventions (hemodynamic support and transfusion threshold adjustment) for those exceeding 50% risk. By integrating real-time laboratory values with clinical indicators, the model allows for dynamic risk assessment that can be readily incorporated into NICU workflows. While demonstrating immediate clinical applicability, further validation across diverse settings and evaluation of long-term neurodevelopmental outcomes remain important next steps.

This research has a few notable drawbacks. First, the single center retrospective design and limited sample size may introduce selection bias and restrict the generalizability of findings. Our model's events-per-variable ratio (EPV = 7.7) falls below the conventional threshold of 10, though we mitigated this through regularization techniques and internal validation. While the 7:3 data split was justified for our cohort, external validation in multicenter studies with larger samples is needed to verify transportability across diverse clinical settings. Additionally, some potentially significant predictors like patent ductus arteriosus were not incorporated, and the study did not assess long term neurodevelopmental outcomes such as cerebral palsy or cognitive deficits. Future research should integrate longitudinal follow-up data to evaluate the model's predictive value for both short term and long term outcomes.

## 5 Conclusions

In conclusion, this study introduces an intuitive and clinically applicable nomogram designed to accurately predict the likelihood of severe IVH in extremely premature newborns. Tailored for healthcare providers—particularly those in frontline pediatric care—this tool leverages readily available patient data to pinpoint infants at elevated risk, allowing for proactive surveillance and early medical intervention. By streamlining risk assessment and supporting personalized treatment plans, the model could significantly mitigate both fatal outcomes and lasting neurological complications, thereby enhancing long term prognosis for these fragile patients.

## Data Availability

The original contributions presented in the study are included in the article/Supplementary Material, further inquiries can be directed to the corresponding author.
